# Supplementary material for: Activation of the CA2-ventral CA1 pathway reverses social discrimination dysfunction in Shank3B knockout mice
Source: Nat Commun. 2023 Mar 29;14:1750. doi: 10.1038/s41467-023-37248-8 (PMC10060401; doi:10.1038/s41467-023-37248-8)
Supplement: Supplementary file 1 — Supplementary Information [file 41467_2023_37248_MOESM1_ESM.pdf]

Supplementary figures

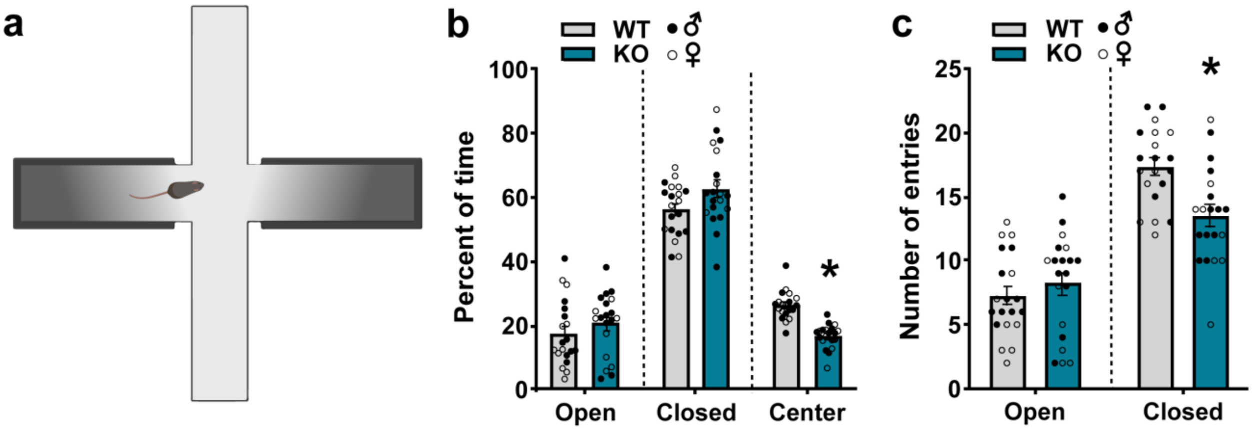

Figure S1. Compared to WT mice, *Shank3B* KO mice did not show increased avoidance behavior in the elevated plus maze. a) Schematic of the elevated plus maze test. **b)** Compared to WT mice, KO mice showed no significant difference in percentage of time in the open arms or closed arms, but a decrease in the percentage of time spent in the center (p = 0.0001). **c)** While the number of entries into the open arms was not different between genotypes, KO mice made fewer entries into the closed arms (p = 0.0011). *p < 0.05; two-sided unpaired t-test (**b, c**) (n = 20 per genotype), see Table S2 for complete statistics. KO = *Shank3B* knockout; WT = wildtype. Image in **a** was created using BioRender.com. Source data are provided as a Source Data file.

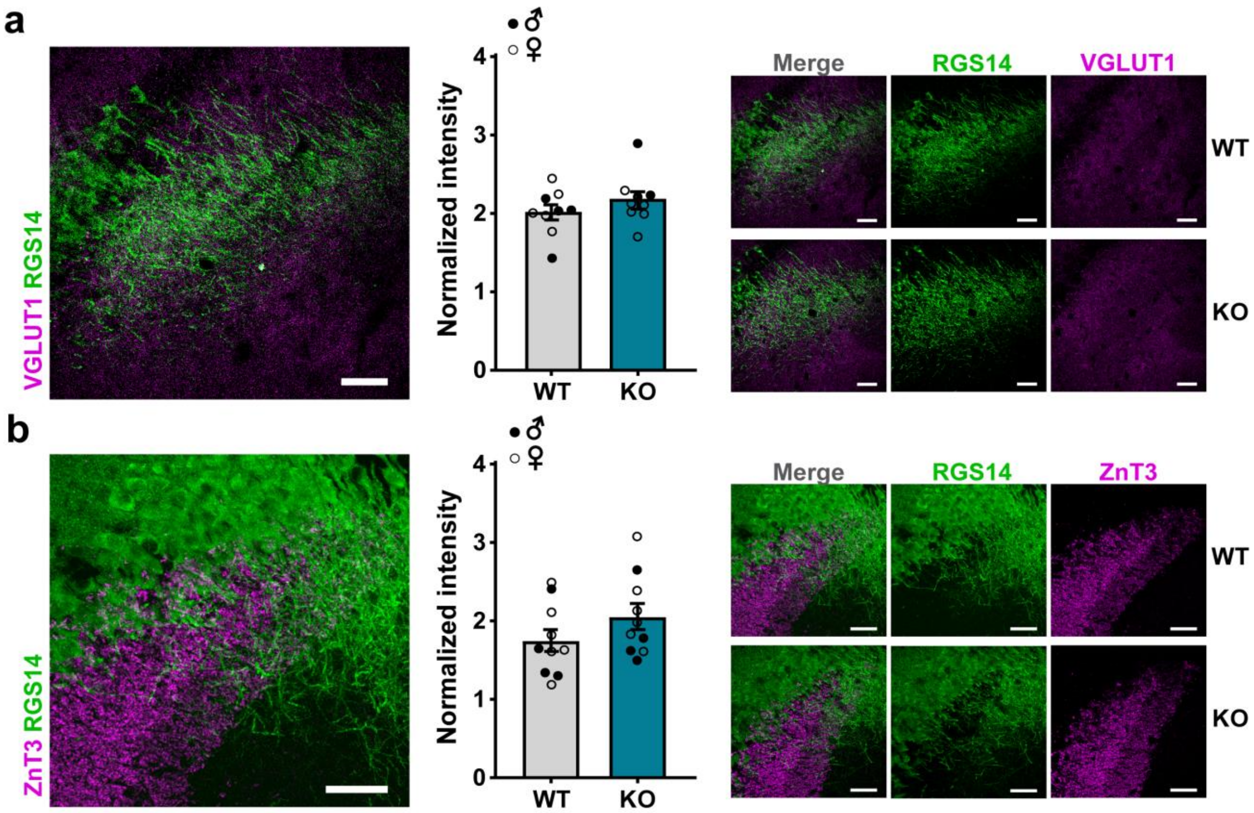

Figure S2. Compared to WT mice, *Shank3B* KO mice showed no difference in VGLUT1+ nor ZnT3+ afferents in the CA2. a) Left: Merged confocal image from a WT mouse of CA2 immunolabeled with RGS14 (green) and excitatory afferent marker VGLUT1 (magenta). Middle: Compared to WT mice, KO mice have similar intensity of VGLUT1+ afferents in the CA2 (n = 9 for each genotype). Right: Merged and single-channel confocal images from WT and KO. **b)** Left: Merged confocal image from a WT mouse of CA2 immunolabeled with CA2 marker RGS14 (green) and mossy fiber marker ZnT3 (magenta). Middle: WT and KO mice had similar intensity of ZnT3+ afferents in the CA2 (n = 10 for each genotype). Right: Merged and single-channel confocal images from WT and KO. Scale bars = 50 µm. See Table S2 for complete statistics. Data are presented as mean $\pm$ SEM analyzed by two-sided unpaired t tests (**a, b**). N = novel; F = familiar; KO = *Shank3B* knockout; WT = wildtype; VGLUT1 = vesicular glutamate transporter, ZnT3 = zinc transporter 3, RGS14 = regulator of G protein signaling 14. Source data are provided as a Source Data file.

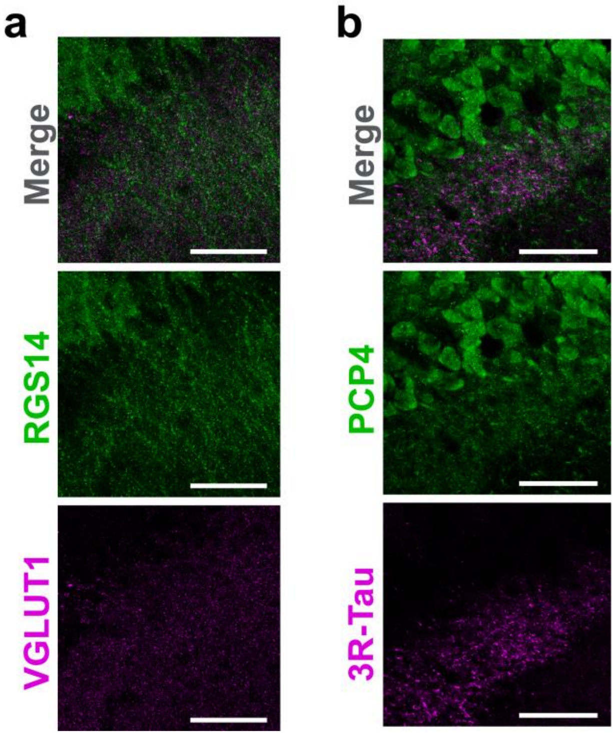

Figure S3. High magnification of VGLUT1+ and 3R-Tau+ afferents in CA2. a) High magnification confocal image of CA2 immunolabeled with RGS14 (green) and excitatory afferent marker VGLUT1 (magenta). Similar staining patterns were observed for all mice included in this experiment**. b)** High magnification confocal image of CA2 immunolabeled with CA2 marker PCP4 (green) and abGC afferent marker 3R-Tau (magenta). Similar staining patterns were observed for all mice included in this experiment Scale bars = 50 µm. PCP4 = Purkinje cell protein 4; RGS14 = regulator of G protein signaling 14; VGLUT1 = vesicular glutamate transporter, ZnT3 = zinc transporter 3; 3R-Tau = 3 repeat Tau isoform.

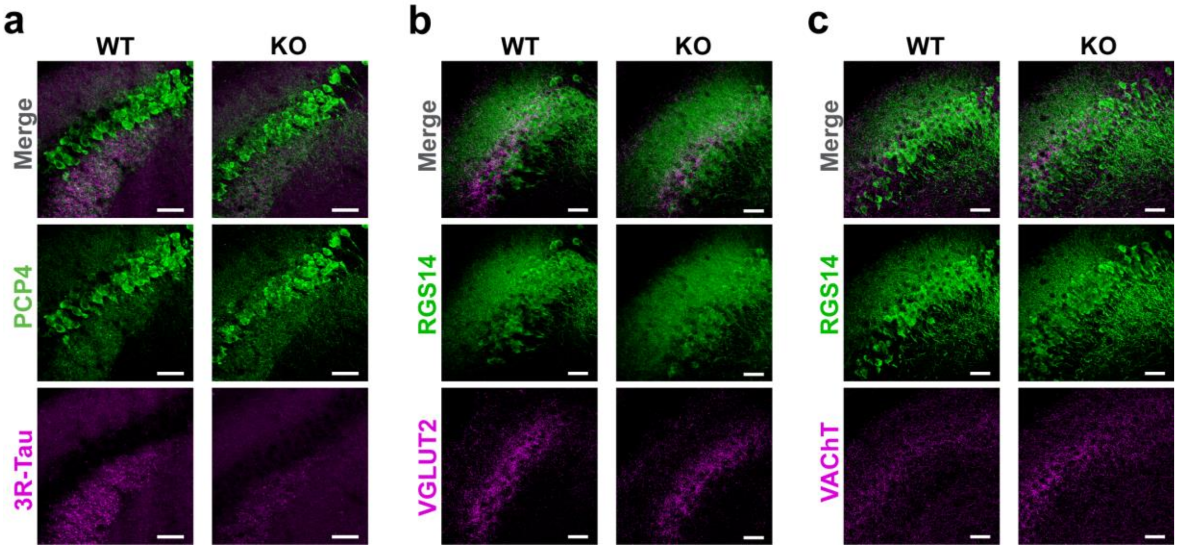

Figure S4. Merged and single-channel confocal images showing mostly typical CA2 afferents, with the exception of 3R-Tau, compared across WT and *Shank3B* KO. a) Merged and single-channel confocal images of CA2 immunolabeled with PCP4 (green) and abGC afferent marker 3R-Tau (magenta) from WT and KO. Similar staining patterns were observed for all mice included in this experiment. **b)** Merged and single-channel confocal images of CA2 immunolabeled with RGS14 (green) and supramammillary nucleus afferent marker VGLUT2 (magenta) from WT and KO. Similar staining patterns were observed for all mice included in this experiment**. c)** Merged and single-channel confocal images of CA2 immunolabeled with CA2 marker RGS14 (green) and cholinergic afferent marker VAChT (magenta) from WT and KO. Similar staining patterns were observed for all mice included in this experiment. Scale bars = 50 µm. WT = wildtype; KO = *Shank3B* KO*;* PCP4 = Purkinje cell protein 4; RGS14 = regulator of G protein signaling 14; 3R-Tau = 3-repeat Tau isoform; VGLUT2 = vesicular glutamate transporter 2; vAChT = vesicular acetylcholine transporter.

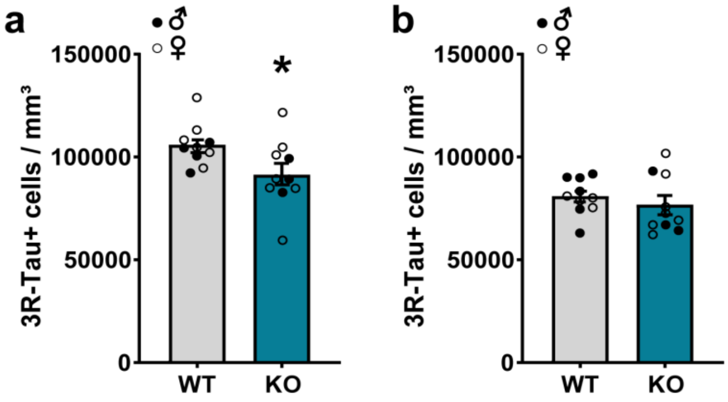

Figure S5. Compared to WT mice, *Shank3B* KO mice had a lower density of abGCs in the suprapyramidal, but not infrapyramidal, blade of the DG. a) Compared to WT mice, KO mice had lower density of 3R-Tau+ cell bodies in the suprapyramidal blade (p = 0.0360), **b)** but not in the infrapyramidal blade, of the DG. *$p < 0.05$ compared to WT mice (n = 10 per group), see Table S2 for complete statistics. Table S2 for complete statistics. *p < 0.05; two-sided unpaired t tests (**a, b**). Data are presented as mean $\pm$ SEM. WT=wildtype; KO = *Shank3B* knockout; 3R-Tau = 3-repeat tau isoform. Source data are provided as a Source Data file.

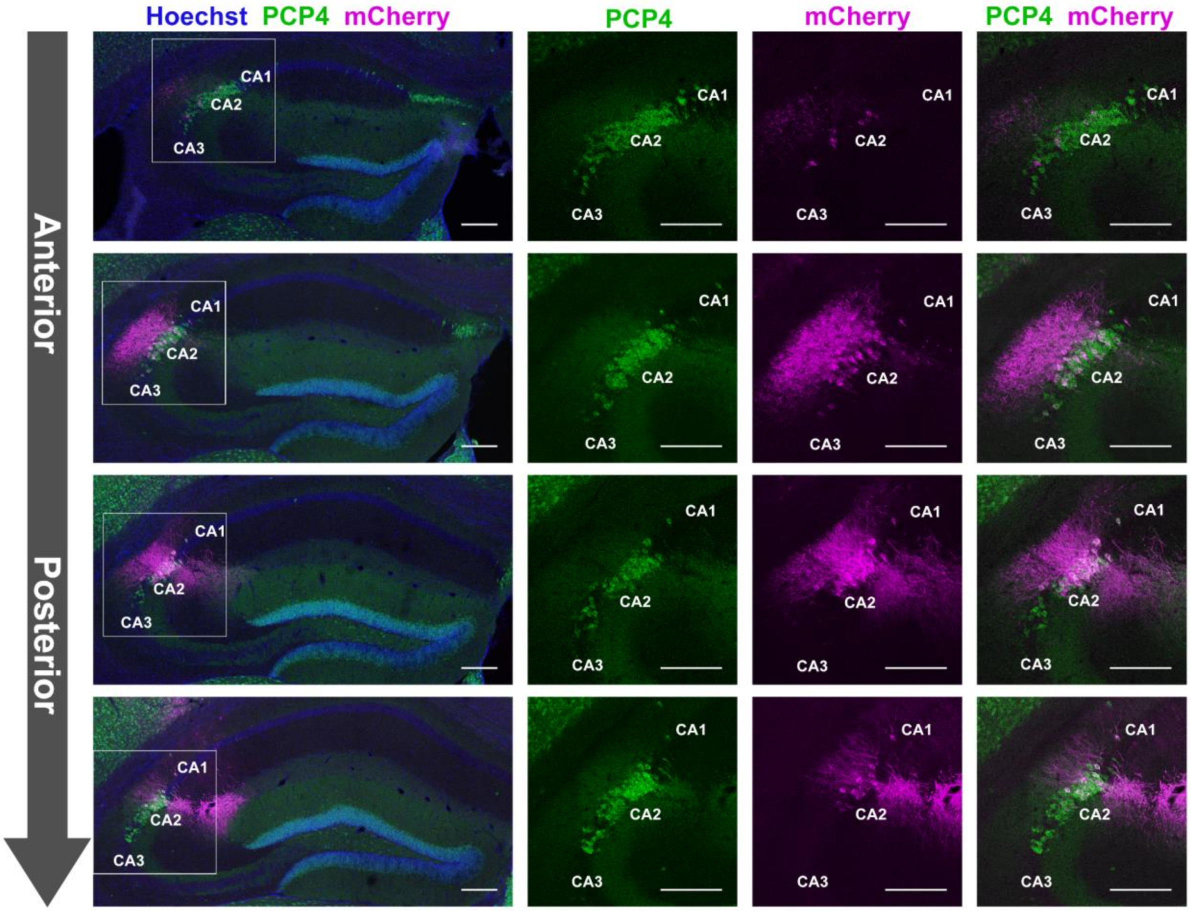

Figure S6. DREADD virus expression was predominantly localized to the CA2 region. Confocal images showing mCherry expression after DREADD virus infection (magenta) in the CA2 immunolabeled with PCP4 (green) and counterstained with Hoechst (blue) at different anterior-posterior levels. Labeling was robust and largely localized to the CA2. Similar viral infection and staining patterns were observed for all mice included in experiments described for Figures 2-4. Scale bars = 200 µm. PCP4=Purkinje cell protein 4.

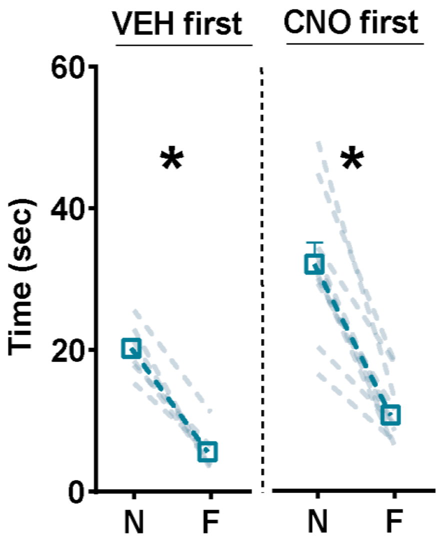

Figure S7. Chemogenetic activation of CA2 excitatory neurons improved social discrimination in *Shank3B* KO mice regardless of the order of CNO administration. Following CNO injections, DREADD virus-infected *Shank3B* KO mice had greater interaction times for N than F in groups that received CNO injections at behavior 1 (p = 0.0001) (n = 10) and groups that received VEH injections at behavior 1 (p = 0.0002) (n = 6). *$p < 0.05$, two-sided paired t-tests. Data are presented as mean ± SEM. See Table S2 for complete statistics. N = novel; F = familiar; VEH = vehicle; CNO = clozapine-n-oxide. Source data are provided as a Source Data file.

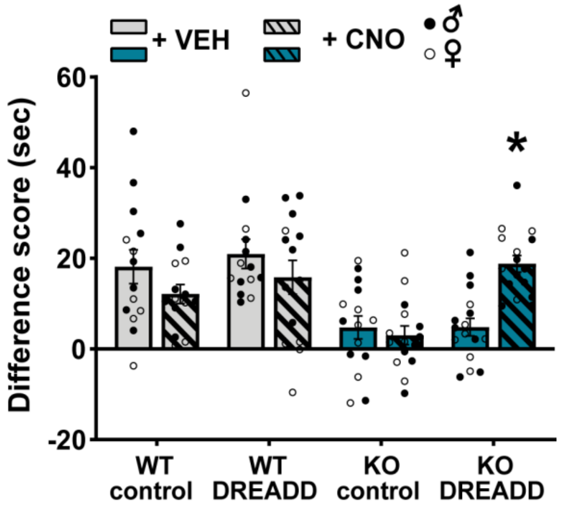

Figure S8. Compared to VEH, CNO treatment improved social discrimination in *Shank3B* KO DREADD virus-injected mice. WT virus groups had high difference scores (N minus F) that were not changed by CNO treatment (WT control virus VEH *vs* CNO). KO control-virus infected mice had low difference scores that were not changed by CNO treatment, while KO DREADD virus-infected mice had higher difference scores following CNO treatment (p = 0.0061) (n = 14 for WT + control virus and DREADD virus, n = 15 for KO + control virus, and n = 16 for KO + DREADD virus). *p < 0.05, three-way repeated measures ANOVA with Bonferroni tests. Data are presented as mean <u>+</u> SEM. KO = *Shank3B* knockout; WT = wildtype; VEH = vehicle; CNO = clozapine-N-oxide; sec = seconds. See Table S2 for complete statistics. Data are presented as mean <u>+</u> SEM. Source data are provided as a Source Data file.

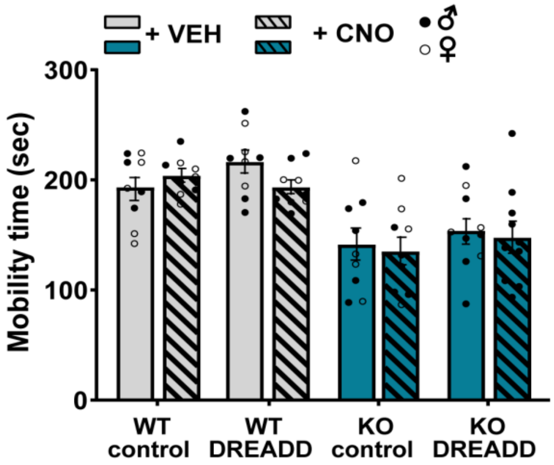

Figure S9. Chemogenetic activation of the CA2 did not alter mobility time during novel mouse exposure. During novel mouse exposure, there was no difference in the time spent mobile between VEH and CNO trials in any of the virus groups. However, there was a decrease in mobility time in KO mice compared to WT mice, regardless of virus group or drug treatment (n = 9 WT control virus, WT DREADD virus, KO control virus; n = 10 KO DREADD virus). Analyzed by three-way repeated measures ANOVA with Bonferroni tests. Data are presented as mean ± SEM. KO = *Shank3B* knockout; WT = wildtype; VEH = vehicle; CNO = clozapine-N-oxide; sec = seconds; DREADD = designer receptors exclusively activated by designer drugs. See Table S2 for complete statistics. Source data are provided as Source Data file.

.

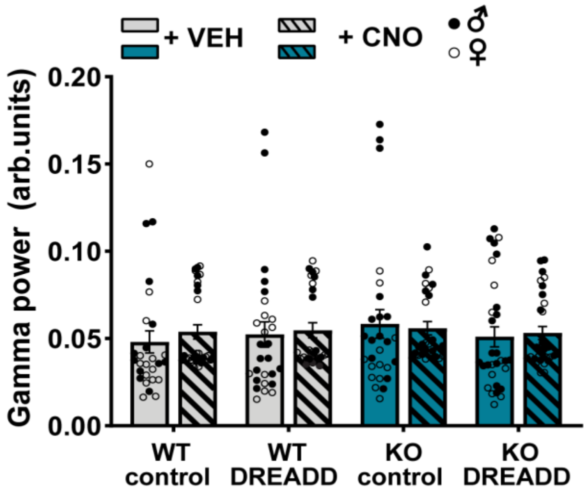

Figure S10. Chemogenetic activation of CA2 excitatory neurons did not affect low gamma power in the vCA1 during bouts of mobility. vCA1 low gamma power (30-55 Hz) was not different during bouts of mobility between WT and KO virus groups injected with VEH or CNO during novel mouse exposure (n = number of mice with 3 electrodes per mouse; 10 WT control; n = 9 WT DREADD; n = 9 KO control; n = 10 KO DREADD). Analyzed by linear mixed effects ANOVA. Data are presented as mean ± SEM. Arb.units = arbitrary units; KO = *Shank3B* knockout; WT = wildtype; VEH = vehicle; CNO = clozapine-N-oxide. See Table S2 for complete statistics. Source data provided in Source Data file.

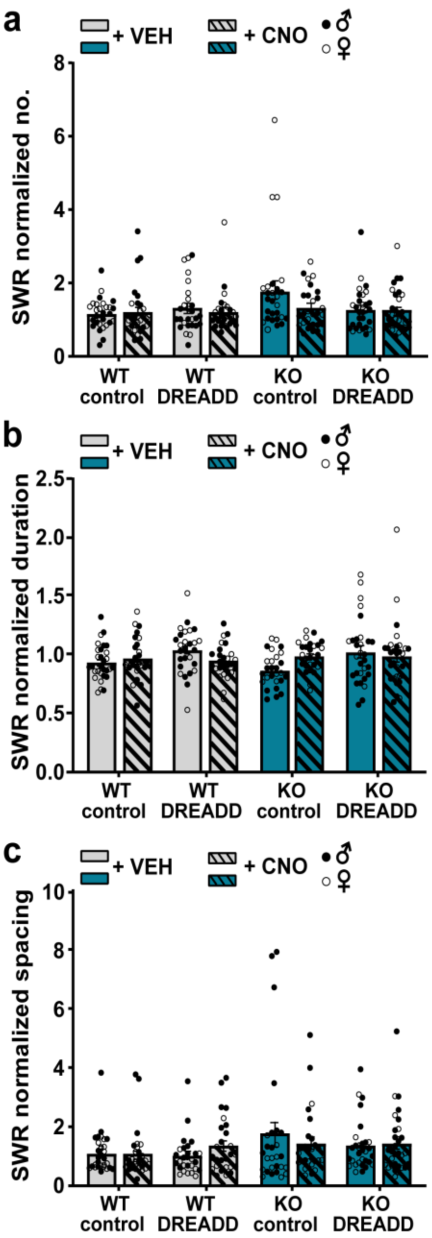

Figure S11. Compared to WT mice, *Shank3B* KO mice showed no difference in SWR numbers, duration, or interval of SWR occurrence during familiar mouse exposure. During the first minute of familiar mouse exposure, there was no difference in **a)** SWR numbers, **b)** SWR duration, or **c)** SWR time interval between genotypes, nor was this changed by CNO-induced activation of the CA2 (A, B, and C, n = number of mice with 3 electrodes for each mouse; n = 10 WT control; n = 9 WT DREADD, KO control; n = 10 KO DREADD). Analyzed by linear mixed effects ANOVA with Tukey comparisons. Data are presented as mean ± SEM. KO = *Shank3B* knockout; WT = wildtype; VEH = vehicle; CNO = clozapine-N-oxide; SWR = sharp wave ripple; no = number. See Table S2 for complete statistics. Source data provided as a Source Data file.

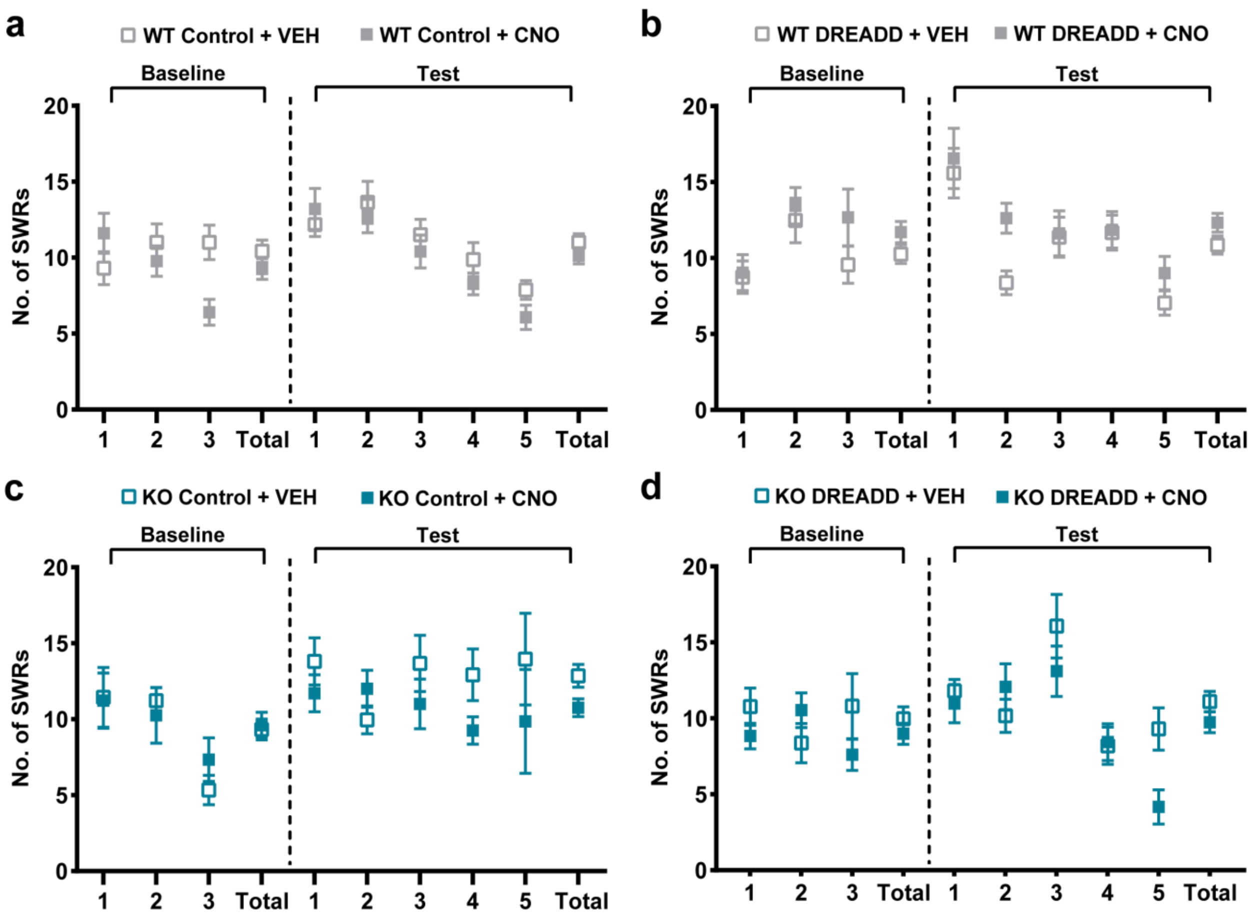

Figure S12. Compared to VEH, CNO treatment showed similar SWR numbers across baseline and familiar testing in WT and *Shank3B* KO mice. Examination of the number of SWRs at each minute bin and total average of the baseline (3 minutes) and familiar test (5 minutes) trial in **a)** WT control virus-injected mice (n = number of mice with 3 electrodes for each mouse; n = 10), **b)** WT DREADD virus-injected mice (n = number of mice with 3 electrodes for each mouse; n = 9) **c)** KO control virus-injected mice (n = number of mice with 3 electrodes for each mouse; n = 10), and **d)** KO DREADD virus-injected mice following treatment with VEH or CNO (n = number of mice with 3 electrodes for each mouse; n=10). Analyzed by linear mixed effects ANOVA with Tukey comparisons. Data are presented as mean ± SEM. KO = *Shank3B* knockout; WT = wildtype; VEH = vehicle; CNO = clozapine-N-oxide; DREADD = designer receptors exclusively activated by designer drugs; no = number. See Table S2 for complete statistics. Source data are provided as a Source Data file.

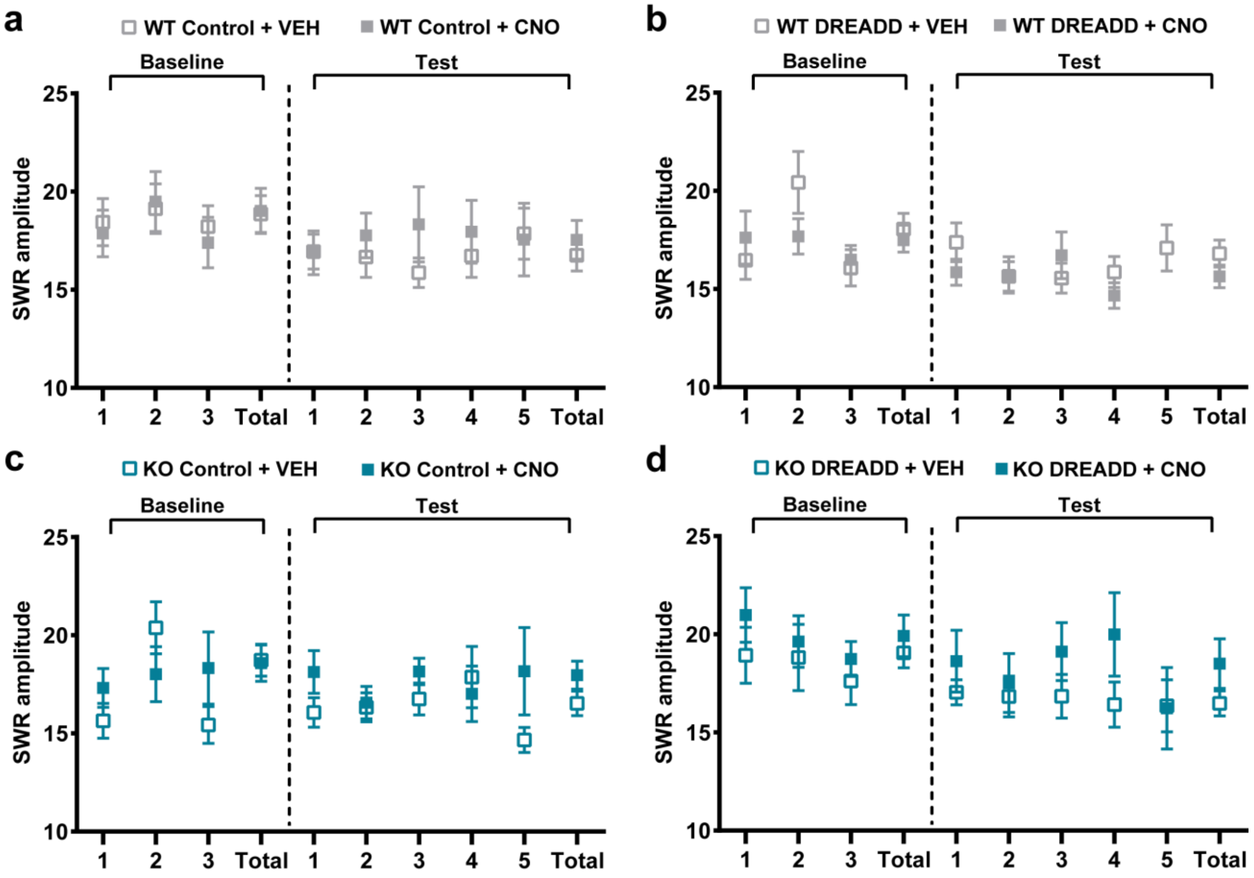

Figure S13. Compared to VEH, CNO treatment showed mostly similar SWR amplitudes across baseline and familiar testing in WT and *Shank3B* KO mice. Examination of the average amplitude of SWRs at each minute bin and total average of the baseline (3 minutes) and familiar test (5 minutes) trial in **a)** WT control virus-injected mice (n = number of mice with 3 electrodes for each mouse; n = 10), **b)** WT DREADD virus-injected mice(n = number of mice with 3 electrodes for each mouse; n = 9) **c)** KO control virus-injected mice(n = number of mice with 3 electrodes for each mouse; n = 10), and **d)** KO DREADD virus-injected mice following treatment with VEH or CNO (n = number of mice with 3 electrodes for each mouse; n = 10). Analyzed by linear mixed effects ANOVA with Tukey comparisons. Data are presented as mean + SEM. KO = *Shank3B* knockout; WT = wildtype; VEH = vehicle; CNO = clozapine-N-oxide; DREADD = designer receptors exclusively activated by designer drugs. See Table S2 for complete statistics. Source data are provided as a Source Data file.

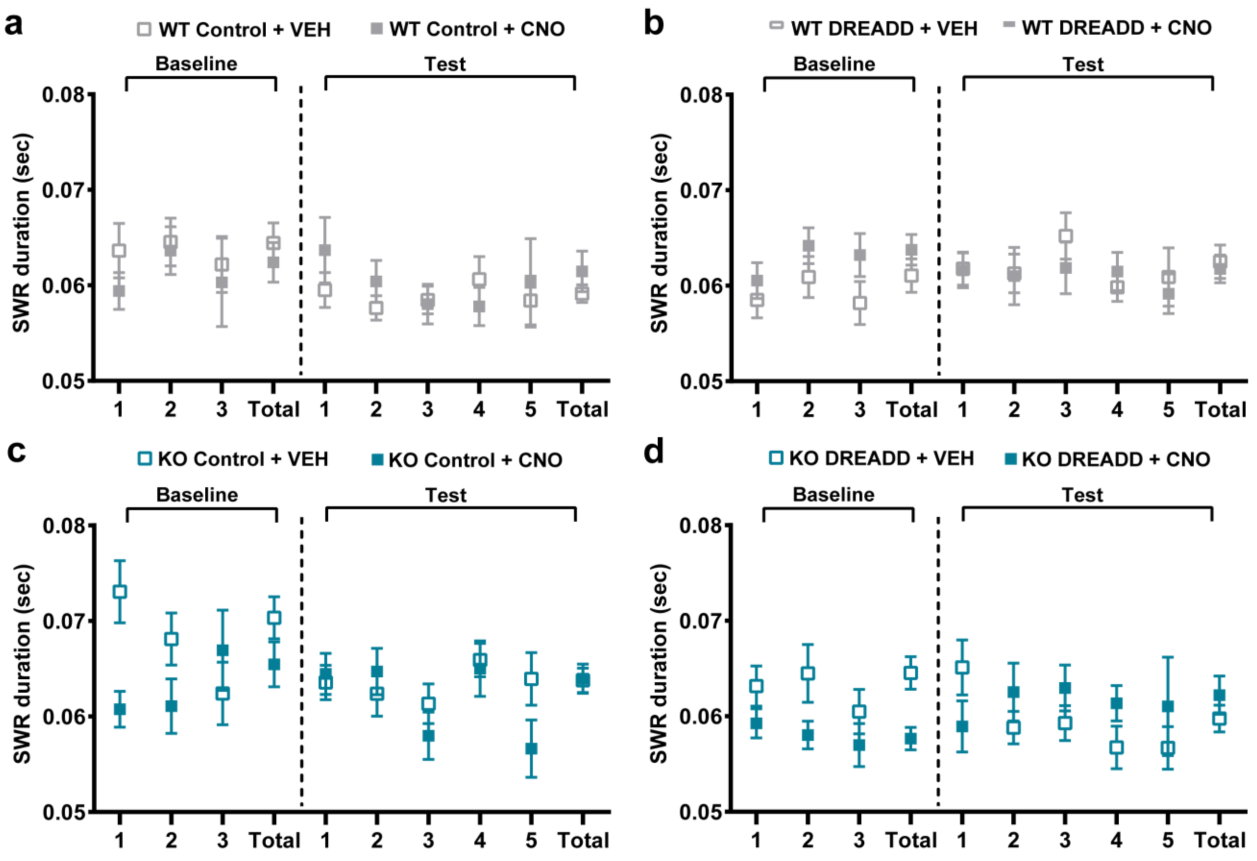

Figure S14. Compared to VEH, CNO treatment showed mostly similar SWR durations across baseline and familiar testing in WT and *Shank3B* KO mice. Examination of the average duration of SWRs at each minute bin and total average of the baseline (3 minutes) and familiar test (5 minutes) trial in **a)** WT control virus-injected mice (n = number of mice with 3 electrodes for each mouse; n = 10), **b)** WT DREADD virus-injected mice(n = number of mice with 3 electrodes for each mouse; n = 9) **c)** KO control virus-injected mice(n = number of mice with 3 electrodes for each mouse; n = 10), and **d)** KO DREADD virus-injected mice following treatment with VEH or CNO (n = number of mice with 3 electrodes for each mouse; n = 10). Analyzed with linear mixed effects ANOVA with Tukey tests. Data are presented as mean ± SEM. KO = *Shank3B* knockout; WT = wildtype; VEH = vehicle; CNO = clozapine-N-oxide; DREADD = designer receptors exclusively activated by designer drugs; no = number. See Table S2 for complete statistics. Source data are provided as a Source Data file.

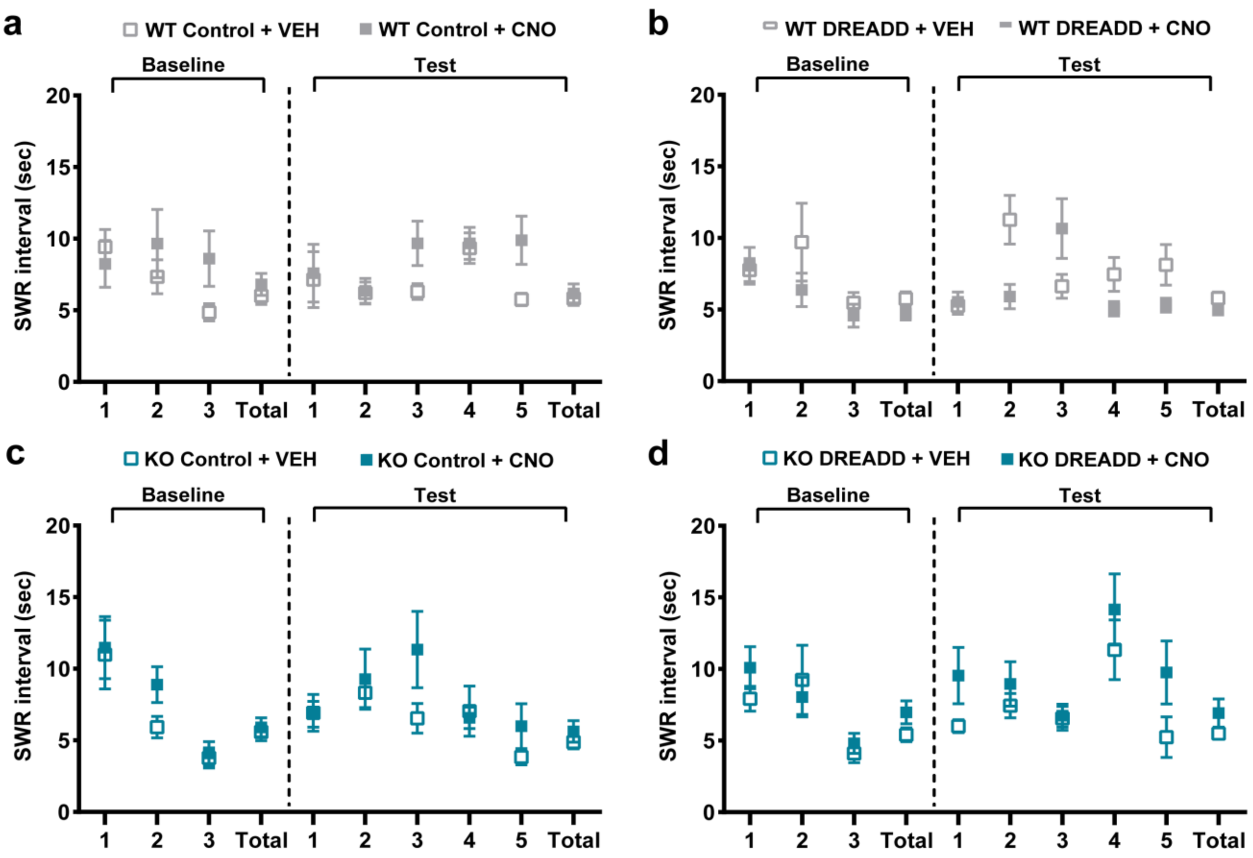

Figure S15. Compared to VEH, CNO treatment showed mostly similar SWR intervals across baseline and familiar testing in WT and *Shank3B* KO mice. Examination of the average time interval of SWR occurrences at each minute bin and total average of the baseline (3 minutes) and familiar test (5 minutes) trial in **a)** WT control virus-injected mice (n = number of mice with 3 electrodes for each mouse; n = 10), **b)** WT DREADD virus-injected mice (n = number of mice with 3 electrodes for each mouse; n = 9) **c)** KO control virus-injected mice(n = number of mice with 3 electrodes for each mouse; n = 10), and **d)** KO DREADD virus-injected mice following treatment with VEH or CNO (n = number of mice with 3 electrodes for each mouse; n = 10). Analyzed by linear mixed effects ANOVA with Tukey tests. Data are presented as mean ± SEM. KO = *Shank3B* knockout; WT = wildtype; VEH = vehicle; CNO = clozapine-N-oxide; DREADD = designer receptors exclusively activated by designer drugs; no = number. See Table S2 for complete statistics. Source data are provided as a Source Data file.

Table S1. Detailed statistics for main figures

Data set	Type of test	Confidence
Figure 1B	two-way repeated measures ANOVA with Bonferroni post hoc comparisons	<u>ANOVA</u><br>trial: $F_{(1.389,31.95)} = 10.33$, $p = 0.0012$<br>genotype: $F_{(1,23)} = 12.18$, $p = 0.002$<br>trial X genotype: $F_{(2,46)} = 9.358$, $p = 0.0004$<br><br><u>post hoc comparisons</u><br>WT N1 *vs* WT F: $p = 0.0001$<br>WT F *vs* WT N2: $p = 0.0112$<br>KO N1 *vs* KO F: $p = 0.9999$<br>KO F *vs* KO N2: $p = 0.1911$<br>WT N1 *vs* KO N1: $p = 0.0009$
Figure 1C	unpaired t-test	N1 minus F: $t_{23} = 5.086$, $p = 0.0001$<br>F minus N2: $t_{23} = 4.055$, $p = 0.000491$
Figure 1E	unpaired t-test	$t_{16} = 0.662$, $p = 0.517$
Figure 1F	unpaired t-test	$t_{16} = 0.730$, $p = 0.476$
Figure 1G	unpaired t-test	$t_{16} = 0.4225$, $p = 0.6783$
Figure 1H	unpaired t-test	$t_{17} = 3.647$, $p = 0.002$
Figure 1I	Mann-Whitney U test	$U_{16} = 21.50$ $p = 0.0984$
Figure 1J	unpaired t-test	$t_{16} = 0.5054$, $p = 0.6202$
Figure 2C	three-way repeated measures ANOVA with Bonferroni post hoc comparisons	<u>ANOVA</u><br>trial: $F_{(1,55)} = 71.93$, $p = 0.0001$<br>genotype: $F_{(1,55)} = 16.87$, $p = 0.0001$<br>virus: $F_{(1,55)} = 1.002$, $p = 0.3213$<br>trial X genotype: $F_{(1,55)} = 26.46$, $p = 0.0001$<br>trial X virus: $F_{(1,55)} = 0.2604$, $p = 0.6119$<br>genotype X virus: $F_{(1,55)} = 0.7023$, $p = 0.4056$<br>trial X genotype X virus: $F_{(1,55)} = 0.2271$, $p = 0.6356$<br><br><u>post hoc comparisons</u><br>WT control virus N *vs* F: $p = 0.0001$<br>WT DREADD virus N *vs* F: $p = 0.0001$<br>KO control virus N *vs* F: $p = 0.9999$<br>KO DREADD virus N *vs* F: $p = 0.9999$
Figure 2D	two-way ANOVA with Bonferroni post hoc comparisons	<u>ANOVA</u><br>genotype: $F_{(1,55)} = 26.46$, $p = 0.0001$<br>virus: $F_{(1,55)} = 0.2604$, $p = 0.6119$<br>genotype X virus: $F_{(1,55)} = 0.2271$, $p = 0.6356$<br><br><u>post hoc comparisons</u><br>WT control virus *vs* KO control virus: $p = 0.0110$<br>WT DREADD virus *vs* KO control virus: $p = 0.0013$

		WT control virus *vs* KO DREADD virus: p = 0.0102<br>WT DREADD virus *vs* KO DREADD virus: p = 0.0011<br>KO control virus *vs* KO DREADD virus: p = 0.9999<br>WT control virus *vs* WT DREADD virus: p = 0.9999
Figure 2E	three-way repeated measures ANOVA with Bonferroni post hoc comparisons	<u>ANOVA</u><br>trial: $F_{(1,55)}$ = 99.39, p = 0.0001<br>genotype: $F_{(1,55)}$ = 1.010, p = 0.3193<br>virus: $F_{(1,55)}$ = 4.029, p = 0.0497<br>trial X genotype: $F_{(1,55)}$ = 1.508, p = 0.2247<br>trial X virus: $F_{(1,55)}$ = 15.11, p = 0.0003<br>genotype X virus: $F_{(1,55)}$ = 3.215, p = 0.0785<br>trial X genotype X virus: $F_{(1,55)}$ = 5.810, p = 0.0193<br><br><u>post hoc comparisons</u><br>WT control virus N *vs* F: p = 0.0002<br>WT DREADD virus N *vs* F: p = 0.0001<br>KO control virus N *vs* F: p = 0.9999<br>KO DREADD virus N *vs* F: p = 0.0001
Figure 2F	two-way ANOVA with Bonferroni post hoc comparisons	<u>ANOVA</u><br>genotype: $F_{(1,55)}$ = 1.508, p = 0.2247<br>virus: $F_{(1,55)}$ = 15.11, p = 0.0003<br>genotype X virus: $F_{(1,55)}$ = 5.810, p = 0.0193<br><br><u>post hoc comparisons</u><br>WT control virus *vs* KO control virus: p = 0.0809<br>WT DREADD virus *vs* KO control: p = 0.0042<br>WT control virus *vs* KO DREADD virus: p = 0.3803<br>WT DREADD virus *vs* KO DREADD virus: p = 0.9999<br>KO control virus *vs* KO DREADD virus: p = 0.0002<br>WT control virus *vs* WT DREADD virus: p = 0.9999
Figure 3C	three-way repeated measures ANOVA with Bonferroni post hoc comparisons	<u>ANOVA</u><br>trial: $F_{(1,57)}$ = 17.09, p = 0.0001<br>genotype: $F_{(1,57)}$ = 12.92, p = 0.0007<br>virus: $F_{(1,57)}$ = 0.02256, p = 0.8811<br>trial X genotype: $F_{(1,57)}$ = 2.434, p = 0.1243<br>trial X virus: $F_{(1,57)}$ = 0.1615, p = 0.6892<br>genotype X virus: $F_{(1,57)}$ = 0.1113, p = 0.7399<br>trial X genotype X virus: $F_{(1,57)}$ = 1.731, p = 0.1935<br><br><u>post hoc comparisons</u><br>WT control virus N *vs* F: p = 0.0270<br>WT DREADD virus N *vs* F: p = 0.3529<br>KO control virus N *vs* F: p = 0.9999<br>KO DREADD virus N *vs* F: p = 0.3151
Figure 3D	two-way ANOVA with Bonferroni post hoc comparisons	<u>ANOVA</u><br>genotype: $F_{(1,57)}$ = 2.434, p = 0.1243<br>virus: $F_{(1,57)}$ = 0.1615, p = 0.6892<br>genotype X virus: $F_{(1,57)}$ = 1.731, p = 0.1935

		<u>post hoc comparisons</u><br>WT control virus *vs* KO control virus: p = 0.2669<br>WT DREADD virus *vs* KO control: p = 0.9999<br>WT control virus *vs* KO DREADD virus: p = 0.9999<br>WT DREADD virus *vs* KO DREADD virus: p = 0.9999<br>KO control virus *vs* KO DREADD virus: p = 0.9999<br>WT control virus *vs* WT DREADD virus: p = 0.9999
Figure 3E	three-way repeated measures ANOVA with Bonferroni post hoc comparisons	<u>ANOVA</u><br>trial: $F_{(1,57)}$ = 38.84, p = 0.0001<br>genotype: $F_{(1,57)}$ = 0.05399, p = 0.8171<br>virus: $F_{(1,57)}$ = 1.358, p = 0.2487<br>trial X genotype: $F_{(1,57)}$ = 3.135, p = 0.0820<br>trial X virus: $F_{(1,57)}$ = 3.302, p = 0.0744<br>genotype X virus: $F_{(1,57)}$ = 1.252, p = 0.2679<br>trial X genotype X virus: $F_{(1,57)}$ = 4.260, p = 0.0436<br><br><u>post hoc comparisons</u><br>WT control virus N *vs* F: p = 0.0024<br>WT DREADD virus N *vs* F: p = 0.0079<br>KO control virus N *vs* F: p = 0.9999<br>KO DREADD virus N *vs* F: p = 0.0005
Figure 3F	two-way ANOVA with Bonferroni post hoc comparisons	<u>ANOVA</u><br>genotype: $F_{(1,57)}$ = 3.135, p = 0.0820<br>virus: $F_{(1,57)}$ = 3.302, p = 0.0744<br>genotype X virus: $F_{(1,57)}$ = 4.260, p = 0.0436<br><br><u>post hoc comparisons</u><br>WT control virus *vs* KO control virus: p = 0.0491<br>WT DREADD virus *vs* KO control: p = 0.0893<br>WT control virus *vs* KO DREADD virus: p = 0.9999<br>WT DREADD virus *vs* KO DREADD virus: p = 0.9999<br>KO control virus *vs* KO DREADD virus: p = 0.0302<br>WT control virus *vs* WT DREADD virus: p = 0.9999
Figure 4C	Linear mixed effects ANOVA with Tukey post hoc comparisons	<u>LME ANOVA</u><br>drug: $F_{(1,186)}$ = 5.5693, p = 0.01931<br>genotype: $F_{(1,34)}$ = 2.1321, p = 0.1534<br>virus: $F_{(1,34)}$ = 0.0939, p = 0.7612<br>drug X genotype: $F_{(1,186)}$ = 4.3871, p = 0.03757<br>drug X virus: $F_{(1,186)}$ = 0.1087, p = 0.7420<br>genotype X virus: $F_{(1,34)}$ = 0.3584, p = 0.5533<br>drug X genotype X virus: $F_{(1,186)}$ = 7.407, p = 0.007114<br><br><u>post hoc comparisons</u><br>WT control virus VEH *vs* CNO: p = 0.5229<br>WT DREADD virus VEH *vs* CNO: p = 0.7284<br>KO control virus VEH *vs* CNO: p = 0.9032<br>KO DREADD virus VEH *vs* CNO: p = 0.0009

Figure 4E	Linear mixed effects ANOVA	LME ANOVA<br>drug: $F_{(1,186)}$ = 3.8653, p = 0.05078<br>genotype: $F_{(1,34)}$ = 0.0591, p = 0.80935<br>virus: $F_{(1,34)}$ = 0.3243, p = 0.57280<br>drug X genotype: $F_{(1,186)}$ = 0.2319, p = 0.6307<br>drug X virus: $F_{(1,186)}$ = 1.3297, p = 0.25033<br>genotype X virus: $F_{(1,34)}$ = 1.9854, p = 0.16790<br>drug X genotype X virus: $F_{(1,186)}$ = 0.3388, p = 0.56121
Figure 4G	Linear mixed effects ANOVA with Tukey post hoc comparisons	LME ANOVA<br>drug: $F_{(1,186)}$ = 3.1207, p = 0.07894<br>genotype: $F_{(1,34)}$ = 0.1055, p = 0.7473<br>virus: $F_{(1,34)}$ = 0.3138, p = 0.5790<br>drug X genotype: $F_{(1,186)}$ = 4.3730, p = 0.03787<br>drug X virus: $F_{(1,186)}$ = 1.1115, p = 0.2931<br>genotype X virus: $F_{(1,34)}$ = 0.1090, p = 0.7433<br>drug X genotype X virus: $F_{(1,186)}$ = 0.0185, p = 0.8919<br><br>post hoc comparisons<br>WT control virus VEH *vs* CNO: p = 0.9901<br>WT DREADD virus VEH *vs* CNO: p = 0.9302<br>KO control virus VEH *vs* CNO: p = 0.0697<br>KO DREADD virus VEH *vs* CNO: p = 0.5196

Table S2. Detailed statistics for supplementary figures

Data set	Type of test	Confidence
Figure S1B	unpaired t-test	Open: t_{38} = 0.992, p = 0.327<br>Closed: t_{38} = 2.016, p = 0.0509<br>Center: t_{38} = 7.485, p = 0.0001
Figure S1C	unpaired t-test	Open: t_{38} = 0.841, p = 0.406<br>Closed: t_{38} = 3.548, p = 0.0011
Figure S2A	unpaired t-test	t_{16} = 1.116, p = 0.2809
Figure S2B	unpaired t-test	t_{18} = 1.399, p = 0.1789
Figure S5A	unpaired t-test	t_{18} = 2.265, p = 0.0360
Figure S5B	unpaired t-test	$_{18}$ = 0.831, p = 0.416
Figure S7	paired t-test	CNO first: t_9 = 8.442, p = 0.0001<br>VEH first: t_5 = 9.0, p = 0.0002
Figure S8	three-way repeated measures ANOVA with Bonferroni post hoc comparisons	<u>ANOVA</u><br>drug: $F_{(1,55)}$ = 0.01956, p = 0.8893<br>genotype: $F_{(1,55)}$ = 23.60, p = 0.0001<br>virus: $F_{(1,55)}$ = 9.263, p = 0.0036<br>drug X genotype: $F_{(1,55)}$ = 8.842, p = 0.0044<br>drug X virus: $F_{(1,55)}$ = 4.387, p = 0.0408<br>genotype X virus: $F_{(1,55)}$ = 1.606, p = 0.2104<br>drug X genotype X virus: $F_{(1,55)}$ = 3.525, p = 0.0658<br><br><u>post hoc comparisons</u><br>WT control virus VEH *vs* CNO: p = 0.9999<br>WT DREADD virus VEH *vs* CNO: p = 0.9999<br>KO control virus VEH *vs* CNO: p = 0.9999<br>KO DREADD virus VEH *vs* CNO: p = 0.0061
Figure S9	three-way repeated measures ANOVA with Bonferroni post hoc comparisons	<u>ANOVA</u><br>drug: $F_{(1,33)}$ = 0.5316, p = 0.4711<br>genotype: $F_{(1,33)}$ = 45.82, p = 0.0001<br>virus: $F_{(1,33)}$ = 1.423, p = 0.2414<br>drug X genotype: $F_{(1,33)}$ = 0.000003578, p = 0.9985<br>drug X virus: $F_{(1,33)}$ = 1.270, p = 0.2679<br>genotype X virus: $F_{(1,33)}$ = 0.1237, p = 0.7273<br>drug X genotype X virus: $F_{(1,33)}$ = 1.177, p = 0.2859<br><br><u>post hoc comparisons</u><br>WT control virus VEH *vs* CNO: p = 0.9999<br>WT DREADD virus VEH *vs* CNO: p = 0.9999<br>KO control virus VEH *vs* CNO: p = 0.9999<br>KO DREADD virus VEH *vs* CNO: p = 0.9999
Figure S10	Linear mixed effects ANOVA	<u>LME ANOVA</u><br>drug: $F_{(1,217)}$ = 0.2369, p = 0.6269<br>genotype: $F_{(1,217)}$ = 0.3577, p = 0.5504<br>virus: $F_{(1,217)}$ = 0.0993, p = 0.7530<br>drug X genotype: $F_{(1,217)}$ = 0.2831, p = 0.5952<br>drug X virus: $F_{(1,217)}$ = 0.0064, p = 0.9362<br>genotype X virus: $F_{(1,217)}$ = 0.9166, p = 0.3394

		drug X genotype X virus: $F_{(1,217)}$ = 0.2611, *p* = 0.6099
Figure S11A	Linear mixed effects ANOVA	LME ANOVA<br>drug: $F_{(1,186)}$ = 2.8895, *p* = 0.09083<br>genotype: $F_{(1,34)}$ = 1.6928, *p* = 0.2020<br>virus: $F_{(1,34)}$ = 0.5803, *p* = 0.4515<br>drug X genotype: $F_{(1,186)}$ = 2.2020, *p* = 0.1395<br>drug X virus: $F_{(1,186)}$ = 0.7378, *p* = 0.3915<br>genotype X virus: $F_{(1,34)}$ = 1.6019, *p* = 0.2142<br>drug X genotype X virus: $F_{(1,186)}$ = 3.2905, *p* = 0.0713
Figure S11B	Linear mixed effects ANOVA with Tukey post hoc comparisons	LME ANOVA<br>drug: $F_{(1,186)}$ = 0.0418, *p* = 0.8383<br>genotype: $F_{(1,34)}$ = 0.0001, *p* = 0.9914<br>virus: $F_{(1,34)}$ = 2.4445, *p* = 0.1272<br>drug X genotype: $F_{(1,186)}$ = 2.3433, *p* = 0.1275<br>drug X virus: $F_{(1,186)}$ = 8.6790, *p* = 0.003631<br>genotype X virus: $F_{(1,34)}$ = 0.1914, *p* = 0.6646<br>drug X genotype X virus: $F_{(1,186)}$ = 0.1104, *p* = 0.7401<br><br>post hoc comparisons<br>WT control virus VEH *vs* CNO: *p* = 0.9114<br>WT DREADD virus VEH *vs* CNO: *p* = 0.2231<br>KO control virus VEH *vs* CNO: *p* = 0.0726<br>KO DREADD virus VEH *vs* CNO: *p* = 0.8577
Figure S11C	Linear mixed effects ANOVA	LME ANOVA<br>drug: $F_{(1,186)}$ = 0.0048, *p* = 0.9448<br>genotype: $F_{(1,34)}$ = 2.1885, *p* = 0.1483<br>virus: $F_{(1,34)}$ = 0.0629, *p* = 0.8036<br>drug X genotype: $F_{(1,186)}$ = 1.1656, *p* = 0.2817<br>drug X virus: $F_{(1,186)}$ = 2.9433, *p* = 0.0879<br>genotype X virus: $F_{(1,34)}$ = 0.3957, *p* = 0.5335<br>drug X genotype X virus: $F_{(1,186)}$ = 0.0019, *p* = 0.9651
Figure S12	Linear mixed effects ANOVA with Tukey post hoc comparisons	LME ANOVA<br>Baseline trial minute 1: all effects n.s.<br>Baseline trial minute 2: drug X virus: $F_{(1,186)}$ = 4.0205, *p* = 0.0464; all other effects are n.s.<br>Baseline trial minute 3: drug X genotype X virus: $F_{(1,186)}$ = 20.9919, *p* = 0.0001; all other effects are n.s.<br>Baseline trial average: drug X genotype X virus: $F_{(1,186)}$ = 6.7848, *p* = 0.009937; all other effects are n.s.<br>Familiar trial minute 1: all effects n.s.<br>Familiar trial minute 2: drug: $F_{(1,186)}$ = 7.1717, *p* = 0008069; all other effects are n.s.<br>Familiar trial minute 3: all effects are n.s.<br>Familiar trial minute 4: drug X virus: $F_{(1,186)}$ = 4.1223, *p* = 0.04375; all other effects are n.s.<br>Familiar trial minute 5: drug X genotype: $F_{(1,186)}$ = 4.5943, *p* = 0.03338; all other effects are n.s.

		Familiar trial average: drug: $F_{(1,186)}$ = 4.0368, *p* = 0.04596; drug X genotype: $F_{(1,186)}$ = 8.1719, *p* = 0.00474; drug X virus: $F_{(1,186)}$ = 4.7293, *p* = 0.03092; all other effects are n.s.<br><br>post hoc comparisons<br>Baseline trial minute 2: all comparisons n.s.<br>Baseline trial minute 3: WT control virus VEH *vs* CNO: *p* = 0.0053; all other comparisons are n.s.<br>Baseline trial average: all comparisons n.s.<br>Familiar trial minute 2: WT DREADD virus VEH *vs* CNO: *p* = 0.0140; all other comparisons are n.s.<br>Familiar trial minute 4: all comparisons n.s.<br>Familiar trial minute 5: all comparisons n.s.<br>Familiar trial average: KO control virus VEH *vs* CNO: *p* = 0.0227; all other comparisons are n.s.
Figure S13	Linear mixed effects ANOVA with Tukey post hoc comparisons	LME ANOVA<br>Baseline trial minute 1: genotype X virus: $F_{(1,32.7)}$ = 6.3348, *p* = 0.01692; all other effects are n.s.<br>Baseline trial minute 2: all effects n.s.<br>Baseline trial minute 3: all effects n.s.<br>Baseline trial average: all effects n.s.<br>Familiar trial minute 1: all effects n.s.<br>Familiar trial minute 2: all effects n.s.<br>Familiar trial minute 3: drug: $F_{(1,185.5)}$ = 4.7977, *p* = 0.02975; all other effects are n.s.<br>Familiar trial minute 4: drug X genotype X virus: $F_{(1,179.7)}$ = 4.3610, *p* = 0.3818; all other effects are n.s.<br>Familiar trial minute 5: all effects n.s.<br>Familiar trial average: all effects n.s.<br><br>post hoc comparisons<br>Baseline trial minute 1: all comparisons n.s.<br>Familiar trial minute 3: all comparisons n.s.<br>Familiar trial minute 4: all comparisons n.s.
Figure S14	Linear mixed effects ANOVA with Tukey post hoc comparisons	LME ANOVA<br>Baseline trial minute 1: drug X genotype: $F_{(1,181.5)}$ = 5.5711, *p* = 0.019320; drug X virus: $F_{(1,181.5)}$ = 6.3811, *p* = 0.01239; all other effects are n.s.<br>Baseline trial minute 2: drug X genotype: $F_{(1,181.0)}$ = 6.9822, *p* = 0.008954; all other effects are n.s.<br>Baseline trial minute 3: all effects n.s.<br>Baseline trial average: drug X genotype: $F_{(1,186)}$ = 6.4953, *p* = 0.01162; all other effects are n.s.<br>Familiar trial minute 1: all effects n.s.<br>Familiar trial minute 2: all effects n.s.<br>Familiar trial minute 3: all effects n.s.<br>Familiar trial minute 4: all effects n.s.

		Familiar trial minute 5: all effects n.s.<br>Familiar trial average: all effects n.s.<br><br>post hoc comparisons<br>Baseline trial minute 1: KO control virus VEH *vs* CNO: $p = 0.0004$; all other comparisons are n.s.<br>Baseline trial minute 2: all comparisons n.s.<br>Baseline trial average: KO DREADD virus VEH *vs* CNO: $p = 0.0216$; all other comparisons are n.s.
Figure S15	Linear mixed effects ANOVA with Tukey post hoc comparisons	LME ANOVA<br>Baseline trial minute 1: all effects n.s.<br>Baseline trial minute 2: drug X virus: $F_{(1,177.8)} = 7.3084$, $p = 0.007529$; all other effects are n.s.<br>Baseline trial minute 3: all effects n.s.<br>Baseline trial average: drug X genotype X virus: $F_{(1,186)} = 4.9182$, $p = 0.2779$; all other effects are n.s.<br>Familiar trial minute 1: all effects n.s.<br>Familiar trial minute 2: drug X genotype: $F_{(1,186)} = 6.1148$, $p = 0.01430$; all other effects are n.s.<br>Familiar trial minute 3: drug: $F_{(1,185.2)} = 11.3620$, $p = 0.0009123$; all other effects are n.s.<br>Familiar trial minute 4: genotype X virus: $F_{(1,33.9)} = 7.1283$, $p = 0.01155$; all other effects are n.s.<br>Familiar trial minute 5: drug X genotype X virus: $F_{(1,167.6)} = 5.8664$, $p = 0.0165$<br>Familiar trial average: all effects n.s.<br><br>post hoc comparisons<br>Baseline trial minute 2: all comparisons n.s.<br>Baseline trial average: all comparisons n.s.<br>Familiar trial minute 2: WT DREADD virus VEH *vs* CNO: $p = 0.0050$; all other comparisons are n.s.<br>Familiar trial minute 3: all comparisons n.s.<br>Familiar trial minute 4: all comparisons n.s.<br>Familiar trial minute 5: all comparisons n.s.
