# Supplementary material for: Activation of the CA2-ventral CA1 pathway reverses social discrimination dysfunction in Shank3B knockout mice
Source: Nat Commun. 2023 Mar 29;14:1750. doi: 10.1038/s41467-023-37248-8 (PMC10060401; doi:10.1038/s41467-023-37248-8)
Supplement: Supplementary file 3 — Reporting Summary [file 41467_2023_37248_MOESM3_ESM.pdf]

nature portfolio

Corresponding author(s): Elizabeth Gould

Last updated by author(s): 3/9/2023

Reporting Summary

Nature Portfolio wishes to improve the reproducibility of the work that we publish. This form provides structure for consistency and transparency in reporting. For further information on Nature Portfolio policies, see our Editorial Policies and the Editorial Policy Checklist.

Statistics

For all statistical analyses, confirm that the following items are present in the figure legend, table legend, main text, or Methods section.

n/a	Confirmed	
☐	☒	The exact sample size (*n*) for each experimental group/condition, given as a discrete number and unit of measurement
☐	☒	A statement on whether measurements were taken from distinct samples or whether the same sample was measured repeatedly
☐	☒	The statistical test(s) used AND whether they are one- or two-sided<br>*Only common tests should be described solely by name; describe more complex techniques in the Methods section.*
☒	☐	A description of all covariates tested
☐	☐	A description of any assumptions or corrections, such as tests of normality and adjustment for multiple comparisons
☐	☐	A full description of the statistical parameters including central tendency (e.g. means) or other basic estimates (e.g. regression coefficient) AND variation (e.g. standard deviation) or associated estimates of uncertainty (e.g. confidence intervals)
☐	☒	For null hypothesis testing, the test statistic (e.g. *F*, *t*, *r*) with confidence intervals, effect sizes, degrees of freedom and *P* value noted<br>*Give P values as exact values whenever suitable.*
☒	☐	For Bayesian analysis, information on the choice of priors and Markov chain Monte Carlo settings
☒	☐	For hierarchical and complex designs, identification of the appropriate level for tests and full reporting of outcomes
☐	☒	Estimates of effect sizes (e.g. Cohen's *d*, Pearson's *r*), indicating how they were calculated

Our web collection on statistics for biologists contains articles on many of the points above.

Software and code

Policy information about availability of computer code

Data collection	IElectrophysiological recordings were collected using a wireless headstage (TBSI, Harvard Biosciences). The data were transmitted to a wireless receiver (Triangle Biosystems) and data were recorded with NeuroWare software version 3.0 (Triangle Biosystems).Confocal images were collected using LAS X software 3.5.6. Data were organized using Microsoft Excel 16.38 spreadsheets .
Data analysis	Optical intensities for afferents (3R-Tau+, ZnT3+, VGLUTl +, VGlUT2+, and VAChT + axons) were analyzed using lmageJ (NIH) version 2.9.0. CA2 areas were analyzed with Stereo Investigator (version 11.03). Electrophysiological recordings were analyzed using Neuroexplorer software version 5.21 (Nex Technologies). A custom Python code was then used to detect sharp-wave ripples (https://github.com/einaraz/SharpWaveRipple). Graph Pad Prism (9.2) was used for graphic visualization and statistical analysis.

For manuscripts utilizing custom algorithms or software that are central to the research but not yet described in published literature, software must be made available to editors and reviewers. We strongly encourage code deposition in a community repository (e.g. GitHub). See the Nature Portfolio guidelines for submitting code & software for further information.

Data

Policy information about availability of data

All manuscripts must include a data availability statement. This statement should provide the following information, where applicable:

- Accession codes, unique identifiers, or web links for publicly available datasets
- A description of any restrictions on data availability
- For clinical datasets or third party data, please ensure that the statement adheres to our policy

The raw electrophysiology data generated in this study is available in the figshare database:
https://figshare.com/projects/Cope_et_al_2023_vCA1_LFP_data_Shank3B/158552.
The raw histology data generated in this study is available in the figshare database at https://figshare.com/projects/Cope_et_al_2023_CA2_Shank3B_ZnT3/159908
https://figshare.com/projects/Cope_et_al_2023_CA2_Shank3B_vGLUT2/159905
https://figshare.com/projects/Cope_et_al_2023_CA2_Shank3B_-_vGLUT1/159851
https://figshare.com/projects/Cope_et_al_2023_CA2_Shank3B_VAChT/159824
https://figshare.com/projects/Cope_et_al_2023_CA2_Shank3B_-_3R-tau/159818
Behavioral data are available from the corresponding author upon request. Source data are provided with this paper.

Human research participants

Policy information about studies involving human research participants and Sex and Gender in Research.

Reporting on sex and gender	*Use the terms sex (biological attribute) and gender (shaped by social and cultural circumstances) carefully in order to avoid confusing both terms. Indicate if findings apply to only one sex or gender; describe whether sex and gender were considered in study design whether sex and/or gender was determined based on self-reporting or assigned and methods used. Provide in the source data disaggregated sex and gender data where this information has been collected, and consent has been obtained for sharing of individual-level data; provide overall numbers in this Reporting Summary. Please state if this information has not been collected. Report sex- and gender-based analyses where performed, justify reasons for lack of sex- and gender-based analysis.*
Population characteristics	*Describe the covariate-relevant population characteristics of the human research participants (e.g. age, genotypic information, past and current diagnosis and treatment categories). If you filled out the behavioural & social sciences study design questions and have nothing to add here, write "See above."*
Recruitment	*Describe how participants were recruited. Outline any potential self-selection bias or other biases that may be present and how these are likely to impact results.*
Ethics oversight	*Identify the organization(s) that approved the study protocol.*

Note that full information on the approval of the study protocol must also be provided in the manuscript.

Field-specific reporting

Please select the one below that is the best fit for your research. If you are not sure, read the appropriate sections before making your selection.

☒ Life sciences ☐ Behavioural & social sciences ☐ Ecological, evolutionary & environmental sciences

For a reference copy of the document with all sections, see nature.com/documents/nr-reporting-summary-flat.pdf

Life sciences study design

All studies must disclose on these points even when the disclosure is negative.

Sample size	Sample sizes were determined according to the standards of the field and previous studies performed in our laboratory.
Data exclusions	Data exclusion criteria were determined during the experimental design stage. Mice with clogged cannulas or lacking viral expression in CA2 were excluded from the study.<br>One mouse was identified as a statistical outlier using the Grubb's test (from the 3R-Tau afferent analyses) and was excluded from the analyses because the value was unusually high suggesting a problem with immunohistochemical staining.
Replication	There were no specific attempts at replication in the manuscript, but the lack of social discrimination in Shank3B KO mice was observed in 3 different experiments (Figures 1, 2, and 3). The restoration of social discrimination abilities with CA2 activation was observed in two experiments (Figures 2 and 3). One control group in Figure 3 did not show a statistically significant reduction in time investigating novel versus familiar mice. All other experiments were not intentionally replicated but within studies, histological staining and viral infection was consistent within groups.

Randomization	All mice were randomly assigned into viral groups. The order of drug administration (CNO or vehicle) was randomized.
Blinding	Animal IDs were coded to prevent potential researcher bias. For all experiments, researchers were blinded to the animal condition throughout the analyses.

Reporting for specific materials, systems and methods

We require information from authors about some types of materials, experimental systems and methods used in many studies. Here, indicate whether each material, system or method listed is relevant to your study. If you are not sure if a list item applies to your research, read the appropriate section before selecting a response.

Materials & experimental systems

n/a	Involved in the study	
☐	☒	Antibodies
☒	☐	Eukaryotic cell lines
☒	☐	Palaeontology and archaeology
☐	☒	Animals and other organisms
☒	☐	Clinical data
☒	☐	Dual use research of concern

Methods

n/a	Involved in the study	
☒	☐	ChIP-seq
☒	☐	Flow cytometry
☒	☐	MRI-based neuroimaging

Antibodies

Antibodies used	Primary antibodies: mouse anti- three microtubule-binding domain tau protein (3R-tau, 1:500, Millipore, Cat# 05-803), rabbit anti-purkinje cell protein 4 (PCP4, 1:500, Sigma-Aldrich, Cat# HPA005792), rat anti-mCherry (1:1000, lnvitrogen, Cat# M11217), mouse anti-regulator of G protein signaling (RGS14, 1:500, UC Davis/NIH NeuroMab, Cat# 75-170), rabbit anti-zinc transporter 3 (ZnT3, 1:500, Alomone labs, Cat# AZf-013), rabbit anti-vesicular glutamate transporter 2 (VGLUT2, 1:500, Synaptic Systems, Cat# 135 403), rabbit anti-vesicular glutamate transporter 1 (VGLUTl, 1:250, lnvitrogen, Cat# 48-2400), rabbit anti-vesicular acetylcholine transporter (VAChT, 1:500, Synaptic Systems, Cat# 139 103)<br><br>Secondary antibodies: donkey anti-rat Alexa Fluor 568 (1:500, Abcam), Cat# ab175475), donkey anti-mouse Alexa Fluor 568 (1:500, lnvitrogen, Cat# A10037) or 647 (,1:500, Milipore, Cat# AP 192SA6MI) or donkey anti-rabbit Alexa Fluor 488 (1:500, lnvitrogen, Cat# A21206)
Validation	All antibodies used are from commercial sources and were chosen based on wide citation in literature.<br>3R-Tau: Tested applications: IHC and WB. Validated by many citations such as PMID 25446721 and PMID 34183768. PCP4: Tested applications: IHC and WB. Validated by the Human Protein Atlas (HPA) project and by many citations such as PMID 33503421, PMID 24336151, PMID 34183768. mCherry: Tested applications: IHC, WB, FC. Validated for IHC by many references on commercial website such as PMID: 32937133, PMID: 34002087, PMID: 31955944. RGS-14: Tested applications: IHC and WB. Validated by many citations such as PMID 28539874 and PMID 24336151. ZnT3: Tested applications: IHC and WB. Validated by many citations such as PMID 31959697 and PMID 24336151. VGLUT2: Tested applications: IHC, IP, ELISA, WB. Validated for IHC by over 60 references on commercial website, such as PMID 33503421. VGLUTl: Tested applications: IHC and WB. According to the commercial website, it was verified by relative expression to insure that the antibody binds to the antigen stated. Validated by many citation such as PMID 32256333. VAChT: Tested applications: IHC, IP, WB. Validated for IHC by over 60 references on commercial website, such as PMID 35173173.

Animals and other research organisms

Policy information about studies involving animals; ARRIVE guidelines recommended for reporting animal research, and Sex and Gender in Research

Laboratory animals	Male and female wild-type and Shank3B KO were used (6 week to 20 month old). Shank3B KO and wildtype littermates were generated by crossing Shank3B heterozygous mice (JAX Stock no. 17688).
Wild animals	The study did not involve wild animals.
Reporting on sex	The study found no sex differences between wildtype and Shank3B KO mice in our initial analyses (reported in the manuscript) and so male and female data were collapsed for all subsequent analyses.
Field-collected samples	The study did not involve samples collected from the field.
Ethics oversight	All animal procedures were approved by the Princeton University Institutional Animal Care and Use Committee and were in accordance with the National Research Council Guide for the Care and Use of Laboratory Animals.

Note that full information on the approval of the study protocol must also be provided in the manuscript.
